# Time-lapse imaging derived morphokinetic variables reveal association with implantation and live birth following *in vitro fertilization*: A retrospective study using data from transferred human embryos

**DOI:** 10.1371/journal.pone.0242377

**Published:** 2020-11-19

**Authors:** Shabana Sayed, Marte Myhre Reigstad, Bjørn Molt Petersen, Arne Schwennicke, Jon Wegner Hausken, Ritsa Storeng

**Affiliations:** 1 Klinikk Hausken, IVF and Gynecology, Haugesund, Norway; 2 Norwegian National Advisory Unit on Women's Health, Oslo University Hospital, Oslo, Norway; 3 BMP Analytics, Consultancy, Viby J, Denmark; University Hospital of Münster, GERMANY

## Abstract

The purpose of this retrospective time-lapse data analysis from transferred preimplantation human embryos was to identify early morphokinetic cleavage variables that are related to implantation and live birth following *in vitro fertilization* (IVF). All embryos were monitored from fertilization check until embryo transfer for a minimum of 44 hours. The study was designed to assess the association between day 2 embryo morphokinetic variables with implantation and live birth based on Known Implantation Data (KID). The kinetic variables were subjected to quartile-based analysis. The predictive ability for implantation and live birth was studied using receiver operator characteristic (ROC) curves. Three morphokinetic variables, time to 2-cells (t2), duration of second cell cycle (cc2) below one threshold and cc2 above another threshold had the highest predictive value with regards to implantation and live birth following IVF treatment. The predictive pre-transfer information has little divergence between fetal heartbeat and live birth data and therefore, at least for early morphokinetic variables up to the four-cell stage (t4), conclusions and models based on fetal heartbeat data can be expected to be valid for live birth datasets as well. The three above mentioned variables (t2, cc2 below one threshold and cc2 above another threshold) may supplement morphological evaluation in embryo selection and thereby improve the outcome of *in vitro fertilization* treatments.

## Introduction

Embryo selection is a central and critical factor when performing embryo transfer during *in vitro fertilization* (IVF). A substantial fraction of human in vitro embryos will not implant after transfer to the uterus [1, 2]. The most widely used method for embryo selection during IVF treatment is the morphological grading of embryos [3]. This method, however, requires embryo observation at specific time points and the information available for the embryologist is relatively limited. Concerns regarding safety and stability of relocating the embryos in and out of the incubator prevent frequent observations. The dependence on the timings of observations as well as the high degree of inter and intra-observer variability in embryo morphological grading, further limits the success of selection schemes [4–6].

With the introduction of time-lapse imaging (TLI) in IVF, continuous surveillance of embryo development by capturing images at defined intervals and focal planes became possible. TLI offers the advantage of viewing cellular activity and embryogenesis in an orderly, flexible and continuous manner. In addition, studies on time-lapse assessment show a high degree of inter and intra-observer agreement, even for novice users [7–10].

Several selection variables identified by TLI are related to embryo development. These variables should address concerns regarding safety, efficacy, and clinical applicability, as documented through prospective, randomized controlled trials. They should demonstrate correlation with clinical outcome, be reproducible and should be based on sound scientific background [11, 12]. Further large-scale retrospective and prospective randomized controlled trials are much desired to validate the efficacy of TLI.

Previous studies have assessed the predictive value of time-lapse biomarkers on various endpoints, such as developmental competence in terms of blastocyst development and implantation, pregnancy prediction and possible prediction of aneuploidy [13–20]. The preferred clinical outcome in IVF is ´the birth of a singleton healthy child at term´ [21–24]. Few studies have however assessed TLI information with live birth as primary endpoint [16, 25, 26].

The aim of our study is to retrospectively identify early developmental morphokinetic variables provided by TLI and evaluate their association with embryo implantation and live birth following IVF treatment.

## Materials and methods

This retrospective observational study was based on 2827 transferred embryos developed from IVF and ICSI fertilized oocytes supplied by 1995 treatments for infertility at Klinikk Hausken, Haugesund, Norway from May 2011 through August 2018. All zygotes/embryos were cultured in the EmbryoScope® time-lapse imaging system (Vitrolife; Sweden) at 37° C and 6% CO_2_ and 5% atmospheric oxygen without humidification and were monitored from fertilization check until embryo transfer (ET) for a minimum of 44 hours.

The analysis utilized Known Implantation Data (KID) [27, 28]. Of the KID treatments, 1163 constituted single embryo transfers (SET) and the remaining 832 constituted double embryo transfers (DET).

The Regional Committee for Medical and Health Research Ethics (REC) (2017/1610) approved the study protocol.

### Ovarian stimulation

Patients underwent midluteal down-regulation using a gonadotropin releasing hormone (GnRH) agonist or antagonist. Ovarian stimulation was planned with daily injections of follicle stimulating hormone (FSH) and/ or human menopausal gonadotropins (hMG), with starting doses based on serum anti-Mullerian hormone levels, antral follicle counts and previous responses to ovarian stimulation. The starting doses varied from 150 IU to 450 IU recombinant FSH. The subsequent doses were adjusted according to follicle development based on ultrasound monitoring. Final follicular maturation was triggered with hCG and /or a GnRH agonist analog, triptorelin acetate, when at least two follicles had a mean diameter of 18mm.

### Oocyte retrieval and insemination

Oocytes were collected by transvaginal ultrasound-guided needle aspiration of the follicles 34–36 hours after trigger. The oocyte cumulus complexes were washed in MOPS (3-(*N*-morpholino) propanesulfonic acid) buffered media with added human serum albumin; GMOPS^TM^ plus (Vitrolife, Sweden). These were then transferred, until denudation /insemination, to bicarbonate buffered medium with added human serum albumin; G-IVF^TM^ plus medium (Vitrolife, Sweden) under a light paraffin oil overlay (Ovoil; Vitrolife, Sweden). Culture dishes were pre-incubated at 37° C with 6% CO_2_ and atmospheric oxygen in humidity set incubators (New Brunswick™ Galaxy® 170 S CO_2_ Incubator). Insemination was either by IVF or by Intracytoplasmic sperm injection (ICSI) depending on the sperm quality as per WHO standards (2010) [29].

The ICSI inseminated oocytes were subsequently transferred to pre-equilibrated 25μl droplets of bicarbonate buffered medium containing hyaluronan and human serum albumin; G-TL ^TM^ plus (Vitrolife, Sweden) and cultured overnight at 37° C with 6% CO_2_ and 5% O_2_. Oocytes allocated for IVF were inseminated with washed and prepared sperm, three to four hours post oocyte retrieval and cultured overnight. The concentration of the insemination drop was set at 1x10^6^ motile spermatozoa/ml. Fertilization was assessed 16–18 hours after insemination for both IVF and ICSI oocytes.

### Embryo culture and time-lapse imaging system

A 12-well EmbryoSlide^TM^ (Vitrolife, Sweden) culture dish was prepared on the day of oocyte retrieval by filling with 25μl of G-TL ^TM^ plus (Vitrolife, Sweden) and 1.5ml oil overlay (Ovoil; Vitrolife, Sweden) and equilibrated overnight at 37° C with 6% CO_2_ and 5% O_2_ in humidity set incubators.

A normally fertilized zygote was evidenced by the presence of two pronuclei and two polar bodies. Following fertilization assessment, these normally fertilized zygotes were transferred to individual wells of the pre-equilibrated EmbryoSlide^TM^ and subsequently placed in the EmbryoScope^TM^ at 37° C with 6% CO_2_ and 5% O_2_ in a non-humidified environment. The zygotes were cultured for a minimum of 44 hours, until the time of either embryo transfer and/or subsequent cryopreservation. Every 15 minutes, the image software of the EmbryoScope^TM^ captures high contrast images in 5–7 focal planes with 200X magnification for each embryo.

### Embryo assessment and time-lapse annotations

Time-lapse images were acquired for each embryo for the duration of the culture period, thereby creating a time-lapse video. For IVF inseminations, the time of insemination was recorded as the time when spermatozoa were added to the oocytes. For ICSI, the time of insemination was entered in the EmbryoScope as the time-point midway through ICSI. All timings were expressed as hours post insemination (hpi) and zygotes were first observed at 16–18 hpi.

Embryo annotations and patient information were stored in the Embryo Viewer software (Vitrolife, Denmark) and in the treatment database IDEAS (Mellowood Medical, Canada). Each patient was identified with a unique registration number and her treatment cycles further had a unique treatment number. Body mass index (BMI), female age at treatment start and primary infertility diagnosis were recorded for each patient.

Time-lapse embryo videos were used to monitor and annotate cell cleavage patterns and nucleation status of the embryos as well as timings of specific cell cycle events. Annotations were done in a sequential manner, starting from the time of first image recording until removal of the EmbryoSlide in order to perform embryo transfer and/ or cryopreservation. Day 2 annotations were done 42–44 hpi. Annotations were done according to a detailed Standard operating procedure implemented in the IVF Laboratory to minimize inter and intra-observer variability in assessments. One embryologist performed annotations independently and this was subsequently double-checked by another embryologist. This procedure was implemented in order to avoid missing annotations and for confirming already annotated images.

### Cleavage kinetic variables

The time-lapse variables annotated to assess their association with the end points included the time of pronuclei fading (tPNf), as well as time of cleavages. The time of cleavage was annotated as the first observed time-point when the newly formed blastomeres were completely separated from each other by confluent cell membranes [30]. The annotated cleavage variables included timings of cell divisions to 2-cell (t2), 3-cell (t3) and 4-cell (t4) expressed as hours post-insemination (hpi). The duration from PN fading to 2- cell stage was denoted as VPN (t2-tPNf). The duration of second cell cycle (cc2 = t3-t2) as well as the synchrony in divisions for the second cell cycle (s2 = t4-t3) were also calculated from these annotated variables as described by Ciray et al. [31].

To assess the association of cleavage kinetics with fetal heartbeat (FHB) and live birth (LB), the above mentioned established TLI morphokinetic variables were used for the analysis. Since 78% of the embryo transfers were performed on day 2 after insemination, the last kinetic parameter utilized in this analysis is t4.

### Embryo selection for transfer and luteal support

Embryo evaluation was based on the established morphological grading of embryos [32] and TLI images. When several good quality embryos were available for transfer, embryo selection was aided by TLI image derived morphokinetics [20]. De-selection criteria were based on cleavage anomalies and nucleation errors [28, 31]. The morphological features of a day-2 embryo were assessed according to the number and symmetry/equality (even or uneven) of blastomere size, degree of fragmentation and presence or absence of a visible nucleus in the blastomeres.

On day 2, good quality embryos were defined as those with four cells, less than 20% fragmentation, high or moderate symmetry, visible nuclei in the individual blastomeres and no signs of multinucleation and cleavage anomalies.

The number of embryos transferred depended on the patient’s previous infertility history, age and embryo quality. Embryos selected for transfer were placed in pre-equilibrated Embryo Glue medium (Vitrolife, Sweden) for a minimum of 10 minutes prior to transfer and embryo transfer was performed under transabdominal ultrasound guidance. Intravaginal progesterone was given as luteal support from the day of oocyte retrieval up to at least the day of positive or negative pregnancy test (16 days after oocyte collection).

### Known implantation data analysis

Known implantation data (KID) analysis was based on the number of gestational sacs, respectively live birth occurrences, matching the number of transferred embryos [27, 28, 33]. This could be either when all the embryos transferred resulted in implantation, defined as KID positive or when none of the transferred embryos resulted in implantation defined as KID negative.

In treatments, where transfer of multiple embryos resulted in single/partial implantation, it was not possible to determine which of the transferred embryo had implanted. The embryos of partial implantations were hence not included in the statistical analysis. A KID value of 1 was given when the outcome was positive and 0 when the outcome for an embryo was negative. The KID values were computed separately for FHB and LB.

### Outcome measures

The primary outcome measures were fetal heartbeat (FHB) and live birth (LB) per embryo transfer. Pregnancy was confirmed by at-home urinary pregnancy test 16 days after oocyte retrieval. A clinical pregnancy was confirmed when a gestational sac with FHB was visible by transvaginal ultrasound done at the clinic, 5 weeks after egg collection. The implantation rate was calculated by dividing the number of gestational sacs with fetal heartbeat by the number of transferred embryos. Early pregnancy loss was tracked as biochemical abortion (before ultrasound showed clinical signs of a pregnancy) or as clinical abortion (after ultrasound has showed clinical signs of a pregnancy).

For the subset of KID embryos, implantation and live birth rates were calculated as 100% x KID positive/ (KID positive + KID negative).

This analysis only included treatments with complete information on ongoing pregnancy and birth status.

Out of the 2827 embryos which are KID based on FHB, the LB KID embryos consist a subset (N = 2769).

### Statistical analyses

All early pre-implantation kinetic time-lapse variables were initially subjected to a quartile-based analysis [20, 30]. Continuous variables such as tPNf, t2, VPN, t3, cc2, t4, t4-t2 and s2 were analyzed. For describing the distribution of the probabilities of implantation and live birth, timings were converted from continuous variables to categorical variables by grouping them into quartiles. The quartile intervals are given in hours and denoted for each variable. This was done to avoid bias that may arise due to differences in total number of embryos in each group. The percentage of embryos that implanted and subsequently resulted in live birth, was assessed for each timing quartile. Fisher’s exact test was used to compare categorical data, including the evaluation of implantation and live birth rates for their association to the early kinetic variables. Results were considered significant at *P* < 0.05.

In order to assess the predictive values of the variables, all combinations of variables were subjected to multivariate logistic regression analysis. The many resulting combinations were tested in automated loops, so that all possible combinations from 1 to 5 concurrent parameters were explored. The only variables kept in the different trials, were these where the null hypothesis that the coefficients were insignificant had a probability below 0.05. Further, only variables whose addition led to a declining Akaike information criterion (AIC) were considered, in order to minimize overfitting.

To further identify the association between cc2 and live births, cc2 timing thresholds defined in two previously published morphokinetic predictive algorithms [20, 34] were tested. Receiver operating characteristic (ROC) curves were used to provide area under the curve (AUC) values to establish the predictive ability of the variables with respect to the end points, implantation, and live birth. An AUC value of 0.5 indicates that there is no predictive ability, while an AUC of 1 indicates perfect predictive ability.

The Mann-Whitney U-test was used to test whether the ROC AUC differs significantly from 0.5. The method of DeLong et.al. [35] was used for comparing the area under two ROC curves.

All statistical analyses were performed using the R statistical software package (R Foundation for Statistical Computing; Vienna, Austria).

## Results

### Patient and embryo outcome data

The present analysis included 1995 ART treatments. The mean age of the patients was 35.5 years (SD 5.3) and mean body mass index was 25.0 (SD 5.1) kg m^- 2^.

Out of the 2827 FHB-KID embryos, 615 implanted (21.8%). Further, 29 FHB-KID treatments resulted in two transfers, but one live birth. Hence, there are fewer LB-KID embryos than FHB- KID embryos. Out of the 2769 LB-KID embryos, 498 resulted in live birth (18.0%).

S1 Table describes the relationship between maternal age at treatment start and FHB-KID and LB-KID grouped into age quartiles. The effect of BMI on FHB-KID and LB-KID is shown in S2 Table.

Considering all embryos, and not only the present KID subset with full LB information available, a total of 2492 treatments and 3723 transferred embryos with recorded outcome resulted in an average implantation rate (FHB) *per embryo* of 27.0% and a success rate *per treatment* (one or more implanted) of 35.3%.

### Analyses of kinetic TLI variables and association to implantation and live birth

PN fading (tPNf) and cell divisions (t2, t3 and t4) on average occur later in the embryos not resulting in live birth than in those with live birth. The IVF embryos exhibited a delay of 0.28 hours in tPNf compared to ICSI embryos. This average time lag is lower than in previously published studies [36, 37]. The analysis does not distinguish between IVF and ICSI embryos.

Table 1 exhibits the association between early morphokinetic variables (tPNf, t2, VPN, t3, cc2, t4, t4-t2, s2) grouped in quartiles for LB-KID. The association between these early kinetic variables grouped in quartiles for LB-KID reveals prominent differences in live birth rates within different quartiles for many of the analyzed variables, especially within quartiles for t2 and tPNf.

**Table 1:** Timing of the kinetic variables from 2769 transferred embryos according to quartiles with LB-KID rates. Occurrences of *P* < 0.001 are marked in bold.

			Q1		Q2		Q3		Q4
Variable	No of embryos	Limit (Hours)	LB-KID rate (%)	Limit (Hours)	LB-KID rate (%)	Limit (Hours)	LB-KID rate (%)	Limit (Hours)	LB-KID rate (%)
tPNf	1703	≤ 22.57	25.4***	22.58–24.46	23.2**	24.46–26.46	21.1	≥ 26.47	8.5***
t2	2764	≤ 25.37	24.8***	25.38–27.20	20.6**	27.21–29.42	17.8	≥ 29.43	8.8***
VPN	1700	≤ 2.33	18.8	2.34–2.66	21.2	2.67–3.00	20.5	≥ 3.01	17.9
t3	2542	≤ 36.08	22.6**	36.09–38.40	22.1**	38.41–40.81	20.2	≥ 40.82	12.5***
cc2	2542	≤ 10.67	19.8	10.68–11.51	22.7**	11.52–12.34	20.5	≥ 12.35	12.1***
t4	2428	≤ 36.99	26.2***	34.00–39.33	22.2	39.34–41.66	19.0	≥ 41.67	12.2***
t4—t2	2426	≤ 11.34	23.1**	11.35–12.30	23.3**	12.31–13.18	19.8	≥ 13.19	13.4***
s2	2426	≤ 0.33	23.7***	0.34–0.66	18.8	0.67–1.17	22.1	≥ 1.18	14.9***

*P < 0.05

**P < 0.01

*** *P* < 0.001. LB-KID: live birth- known implantation data. Variables grouped in quartiles: Q1, Q2, Q3 and Q4.

All annotations had missing values, least for t2 with only 5 annotations missing, and most for VPN with 1069 annotations missing.

S3 Table shows the association between the same variables as for Table 1, grouped in quartiles for FHB-KID. The overall pattern is very similar betweenfor LB-KID and FHB-KID data as seen when comparing Table 1 and S3 Table.

For all cleavage timings assessed, embryos whose cleavage was completed in the first two quartiles (Q1 and Q2) displayed the highest rates for FHB-KID and LB-KID. For most cleavage times, there also is a significant difference in both FHB-KID and LB-KID rates between the embryos in the first quartile (Q1) and the fourth quartile (Q4). For t2, the LB-KID rate decreases from 24.8% in the first quartile (Q1) to 8.8% in Q4 (Table 1). Likewise regarding t2 for FHB-KID rates, this significantly drops from 28.3% in Q1 to 12.1% in Q4 (S3 Table).

For the FHB-KID and LB-KID analyses based on the t2 variable, 5 embryos were missing t2 annotations. These missing annotations were a result of normally fertilized zygotes exhibiting PN fading, but lacking subsequent cell divisions. All variables in Table 1 have occurrences of missing observations. These observations were, however, not purposefully omitted. For instance, some of the PN fading events may have occurred before the embryos were moved to the EmbryoScope, and some of the embryos may have cleaved to four- cells after embryo transfer.

Among TLI variables that are derived as an interval, the duration of the second cell cycle (cc2) displays the most prominent differences in both FHB-KID and LB-KID rates between the different quartiles. The embryos in the second (Q2) quartile had distinctly higher KID rates than those in the fourth (Q4) quartile (22.7% vs. 12.1% LB rates respectively) (Table 1).

Furthermore, in order to obtain possibly suited thresholds for cc2 based on FHB-KID and LB-KID analyses, timing thresholds (Table 2) were derived both from the EEVA model, second version (cc2<9.33h, cc2>11.45h or cc2>12.65h) [34] as well as from Meseguers model (cc2>11.9h) [20]. Utilising these model thresholds for cc2, revealed a pronounced reduction in FHB-KID and LB-KID rates for both the highest and lowest cc2 values, as shown in Table 2. This table had 230 FHB-KID and 227 LB-KID embryos missing cc2 annotations due to missing occurrences of t2 and/or t3.

**Table 2:** Known implantation data rates for FHB-KID and LB-KID by four different time frames for cc2. Each timeframe represents the cc2 response for a published TLI model.

Classifier cc2 (Hours)	No. of embryos	FHB-KID rate (%)	No. of embryos	LB-KID rate (%)
None	2827	21.8	2769	18.0
cc2 < 9.33	199	10.1^a^	195	8.2^b^
EEVA model [34] short cycle				
cc2 > 9.33	2398	24.3^a^	2347	20.1^b^
EEVA model [34] short cycle				
cc2 > 12.65	501	14.8^c^	490	12.0^d^
EEVA model [34] long cycle (1)				
cc2 < 12.65	2096	25.2^c^	2052	20.9^d^
EEVA model [34] long cycle (1)				
cc2 > 11.45	1335	21.7^e^	1301	17.6^f^
EEVA model [34] long cycle (2)				
cc2 < 11.45	1262	24.7^e^	1241	20.9^f^
EEVA model [34] long cycle (2)				
cc2 > 11.9	959	18.8^g^	937	15.2^h^
Messeguer [20]				
cc2 < 11.9	1638	25.8^g^	1605	21.6^h^
Messeguer [20]				

a, b, c, d, g, h: *P* < 0.001; f: *P* < 0.05; e: NS (not significant)

FHB-KID: fetal heart beat implantation rate; LB-KID: live birth- known implantation data; cc2: duration of second cell cycle.

### Quantifying the predictability of implantation and live birth

The predictive power of continuous variables for successful implantation and live birth was assessed by the Area under the curve (AUC) as shown in Table 3. AUC was also used to assess the ability of continuous variables to predict LB-KID for single embryo transfer (SET) and dual embryo transfers (DET), respectively (S4 Table). There were no statistically significant ROC AUC differences between FHB/LB and SET/DET, respectively.

**Table 3:** AUC values to evaluate the ability of continuous TLI variables to predict FHB-KID and LB-KID.

TLI Variable	No. of embryos	AUC (FHB-KID)	No. of embryos	AUC (LB-KID)
tPNf	1747	0.609***	1703	0.618***
t2	2822	0.608***	2764	0.615***
VPN	1744	0.506	1700	0.509
t3	2597	0.568***	2542	0.575***
cc2	2597	0.532*	2542	0.539**
t4	2482	0.590***	2542	0.595***
t4—t2	2479	0.568***	2426	0.567***
s2	2479	0.552***	2426	0.545*

*P < 0.05

**P < 0.01

***P < 0.001.

AUC: Area under the curve. TLI: time-lapse imaging. LB-KID: live birth.

For embryo transfers resulting in live births, tPNf and t2 had AUC values of 0.618 and 0.615 respectively (Table 3) and hence had the best predictive abilities for single variables.

However, there are several tPNf occurrences that precede the TLI incubation, making tPNf less suited as a general predictive variable.

In order to assess the predictive values of the morphokinetic variables, all combinations of possible predictive variables were subjected to multivariate logistic regression analysis with the use of LB-KID. The model yielding the largest AUC value of 0.640 (95% CI 0.615–0.665) utilized three parameters: t2 as continuous variable, the categorical condition cc2<9.33 h and the categorical condition cc2>12.65.

**Fig 1:**
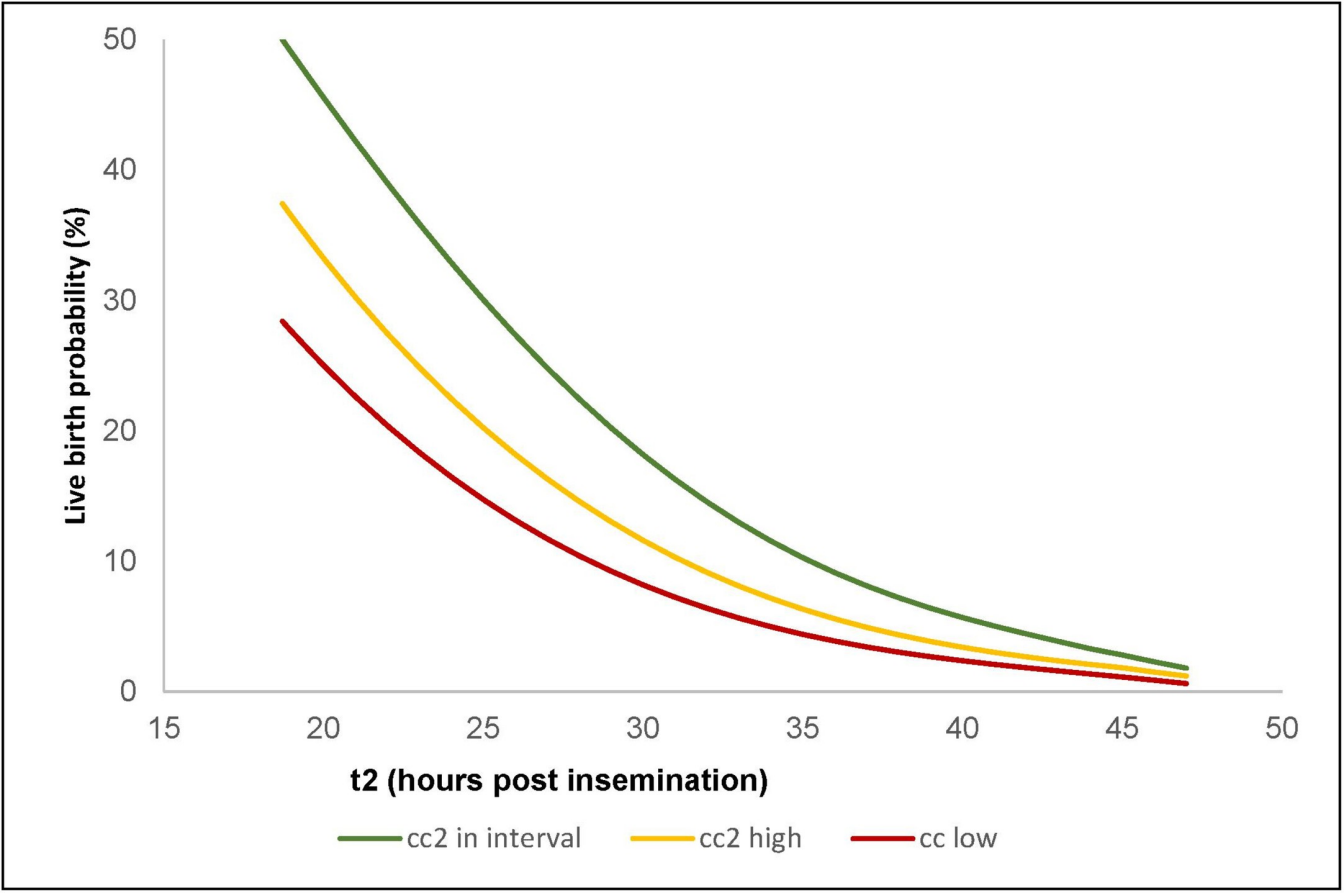
Probability of live birth predicted by logistic regression. The red curve shows the estimated LB probability when cc2<9.33 hours. The yellow curve shows the probability when cc2>12.65 hours. The green curve shows the LB probability when cc2 is between 9.33 and 12.65 hours.

**Fig 2:**
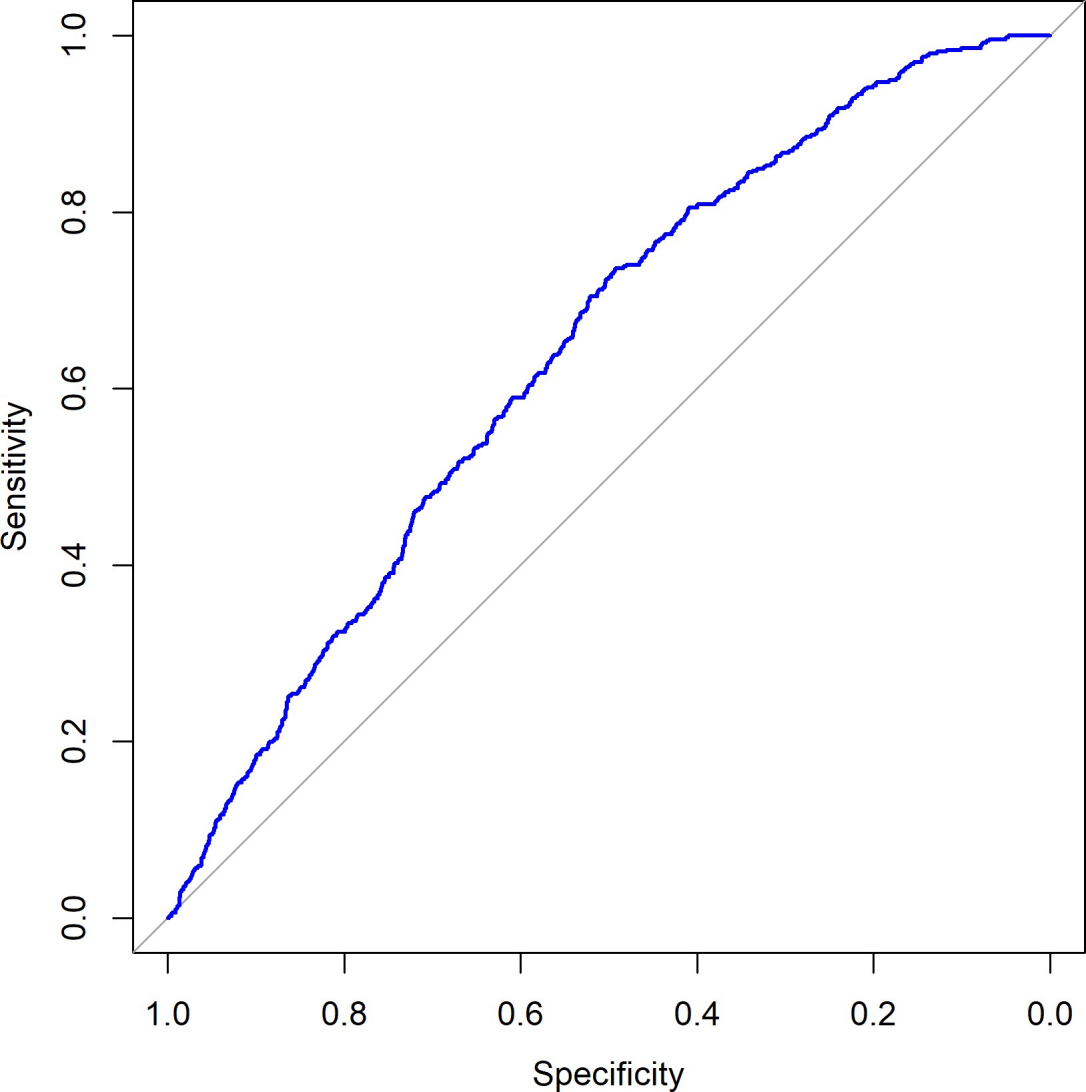
Receiver operating characteristic (ROC) curve. The live birth occurrence is used to visualize predictive properties of the logistic regression model. AUC = 0.640.

Fig 1 shows the probability of live birth as predicted by this three-parameter logistic regression model for all LB-KID transfers, and Fig 2 shows the ROC curve for this model.

The corresponding AUC values for the same three parameters, but based on FHB-KID data is 0.637 (95% CI 0.613–0.660).

The logistic regression model had AUC = 0.632 (95% CI 0.599–0.665) for SET LB data and AUC = 0.624 (95% CI 0.579–0.669) for DET LB data.

## Discussion

This retrospective study evaluates the association of early TLI variables with implantation and live birth rates following IVF treatment. The study demonstrated that early morphokinetic variables serve as valuable biomarkers in predicting an embryo’s potential for implantation and live birth. Three kinetic variables, t2, cc2 below one threshold and cc2 above another threshold provide the most predictive pre-transfer information for both implantation and live birth probabilities when utilized in a logistic regression model. It is further shown that the predictive pre-transfer information has little divergence between FHB and LB data. Therefore, at least for early morphokinetic variables up to t4, conclusions and models based on FHB data can be expected to be valid for LB datasets as well.

The majority of the analyzed embryo transfers were performed on day 2 and it was therefore imperative to find early preimplantation biomarkers that can predict implantation and live births. Prolonged culture to blastocyst does increase the pregnancy rates in IVF cycles if sufficient embryos are available [38]. However, there is always the risk of cancelling a treatment cycle due to lack of blastocysts for transfer, especially in cycles with few available oocytes. Additionally, the concerns of long-term blastocyst culture on genomic imprinting have led to some reports recommending transfer of early-stage embryos [39–41].

The exact timing of the fertilization event is unknown, and further the time between insemination and actual fertilization can, on average, be expected to be lower for ICSI relative to IVF. In our analysis, the IVF embryos exhibited a delay of 0.28 hours in tPNf compared to ICSI embryos. This time lag is considerably lower than in previously published studies [36, 37].

The inclusion of both IVF and ICSI cycles in our study and the stochastic delay from insemination to fertilization makes it difficult to define the exact time point of sperm entry and hence the variables tPNf, t2, t3 and t4 have limitations regarding LB and implementation predictors. However kinetic parameters, such as cc2, represent an interval, whereby the stochastic issue of the time from insemination to fertilization is absent and hence not subject to this kind of uncertainty.

Early cleavage has been an established developmental competence marker in standard morphology selection schemes [42–44]. Additional morphokinetic information obtained via TLI shows a lower implantation and live birth potential for embryos that cleaved after 30 hours(t2>30hrs) [1]. This is in accordance with our data analysis for t2.

Our study demonstrates that morphokinetic variables can serve as valuable biomarkers in predicting an embryo’s potential for implantation and live birth. Cleavage patterns and timings also reflect an embryo’s cytoplasmic and nuclear capabilities [45–47]. Normal cleavage patterns may be indicative of a functional cytoskeleton, a streamlined fertilization related activation event such as Ca2+ oscillation, functional mitochondria as well as a high-quality nuclear apparatus [48]. Several factors could play a role in the occurrence of cleavage aberrations and these delayed and aberrant division patterns may indicate DNA damage or chromosomal segregation errors that can activate some of the cell cycle check points and halt the cell cycle progression, even leading to aneuploidy [49, 50]. The duration of the cell cycle has been established to be approximately around 10–12 hours, enough to undergo two consecutive cytokineses and replication of the whole cell genome [28]. In concordance with this, the present study exhibits negative effects on implantation and live births for first cell cycle shorter than 9.33 hours.

Most attempts to predict implantation from embryo features display comparatively modest AUC values [33, 51, 52]. Having investigated four early morphokinetic variables (tPNf, t2, t3 and t4) and all their combinations, the predictive ability for the regression model using variables t2 and cc2 (AUC = 0.640) is at the same levels as in other studies of model-derived predictions.

This study did not address whether day 3 –day 6 embryo transfers would have benefitted from the additional time-lapse variables available, as there were too few of these transfers to make separate logistic regression models for these cases.

There are probably two major factors that hinder a substantial AUC value for any TLI study of this kind. Foremost there is a preselection bias, due to an embryo selection bias based on established morphological criteria as well as de-selection bias based on established TLI indicators, most prominently direct cleavage [28]. If, hypothetically, this bias was not present, the AUC value would be expected to be substantially higher than for the present logistic regression model. Another important factor that hinders a high predictive capability in terms of AUC, is that the percentage of patients not having a receptive endometrium at the time of the transfer may be quite high [23, 24, 53, 54].

The analysis has to a large extent relied on quartile analyses. This kind of analysis provides a good overview and condenses information to a level that is comprehensible. On the other hand, a quartile analysis only reveals a part of the full picture. Further, embryos may move between quartiles during development. These limitations should be kept in mind when concluding on the analytical findings.

The population of infertile couples treated in our clinic is heterogenous and presents a multitude of infertility diagnoses, BMI values and ages. Therefore, the developed regression is valuable for analytical purposes presently. However, any model of this kind should be tested and validated on independent data before applied to clinical use. During the study period, no major changes were implemented in the IVF laboratory with regards to culture conditions or trained personnel. However, it is also important to consider that during the study period, minor changes to stimulation protocols and fine tuning of embryo selection criteria could have occurred.

In order not to discard a substantial proportion of the embryo data for the analysis, the KID [20, 33, 54] procedures were utilized. While typically providing a dataset that is substantially larger than if only single embryo transfer (SET) data were used, the use of KID to some degree imposes statistical bias on the data. Known implantation rates are typically lower than the implantation rates calculated for the complete patient population as partial implantations are excluded and this in turn results in overrepresentation of non-implantations [28]. This treatment category can skew the data by the exclusion of these partially positive treatments [55]. In contrast, all negative treatments are included, regardless of how many embryos are transferred. Further, the double embryo transfer (DET) data are not truly independent, as the two sibling embryos will always have the same endpoint status in order to provide KID information [13, 55].

One could therefore argue, that only SET embryos should be considered for retrospective studies [55] implying that data analysis would have to await a sufficiently large amount of SET. For retrospective data this might cause a switch from one kind of bias to another possibly even larger bias, though. In most clinics, both one and/or two embryos are transferred. In these clinical settings, there can be a substantial bias generated from a higher probability to transfer more than one embryo to the patients considered having lowered implantation probability.

Liu et al. [55] assumes a concerning overestimation of predictive power when using a KID dataset rather than only SET when validating time-lapse models. In the present study, it was investigated whether this tendency could be confirmed. However, as S3 Table shows, there are no statistically significant differences in predictive power in terms of AUC between SET and DET data. From a theoretical viewpoint, one may also question such a tendency, because the DET data could be expected to be the most stringent data, and therefore be expected to have a better predictive capability. A full clarification of this important issue may have to await a dataset substantially larger than even the present dataset.

The presumably fundamental statistical challenge for all IVF studies, both retrospective and prospective, is the preselection bias underlying the data foundation. This preselection will invariably create considerable bias, making it challenging to identify the underlying patterns clearly.

## Conclusion

The logistic regression model with the highest explanatory value utilized t2 and further, cc2 below one threshold and cc2 above another threshold (9.33, 12.65 hours). These three variables, one continuous and two categorical, provide the most predictive pre-embryo transfer information for both implantation and live birth. We also conclude, based on our study, that models derived from fetal heartbeat data can be expected to be valid also for live birth outcomes, at least for early morphokinetic parameters. This, we believe, will allow time-lapse users to derive their own independent morphokinetics algorithms based on fetal heartbeat data and subsequently validate this for live birth data. In light of the predictive abilities of the above mentioned TLI variables, we recommend supplementing morphological assessment of embryos with morphokinetic information. This may augment embryo selection strategies and thereby enhance outcomes of IVF treatments.

## Supporting information

S1 Table The relationship between age (years) and KID rates (FHB, LB) grouped in age quartiles. (DOCX)

S2 Table The relationship between BMI and KID-rate (FHB, LB) grouped in BMI quartiles. (DOCX)

S3 Table Timing of the kinetic variables from 2827 transferred embryos according to quartiles with FHB-KID rates. Ocurrences of *P* < 0.001 are marked in red.
(DOCX)

S4 Table AUC values to evaluate the ability of continuous TLI variables to predict LB-KID for SET and DET. (DOCX)
