# Supplementary material for: Time-lapse imaging derived morphokinetic variables reveal association with implantation and live birth following in vitro fertilization: A retrospective study using data from transferred human embryos
Source: PLoS One. 2020 Nov 19;15(11):e0242377. doi: 10.1371/journal.pone.0242377 (PMC7676704; doi:10.1371/journal.pone.0242377)
Supplement: S1 Table — (DOCX) [file pone.0242377.s001.docx]

**S1 Table. The relationship between age (years) and KID rates (FHB, LB) grouped in age quartiles.**

|  | **FHB-KID**  **No of embryos** | **Age Limit (Years)** | **FHB-KID rate (%)** | **LB-KID**  **No of embryos** | **Age Limit**  **(Years)** | **LB-KID**  **rate (%)** |
| --- | --- | --- | --- | --- | --- | --- |
| Q1 | 707 | ≤ 31.46 | 37.5*** | 692 | ≤ 31.43 | 35.0*** |
| Q2 | 708 | 31.47 - 35.98 | 28.5*** | 693 | 31.44 - 36.00 | 23.8*** |
| Q3 | 707 | 35.99 - 39.96 | 16.6*** | 692 | 36.00 - 39.98 | 13.1*** |
| Q4 | 705 | ≥ 39.97 | 4.4 *** | 692 | ≥ 39.99 | 4.1*** |

****P* < 0.01

Age quartiles : Q1, Q2, Q3 and Q4. FHB-KID: fetal heart beat implantation rate. LB-KID: live birth rate.
