# Supplementary material for: Time-lapse imaging derived morphokinetic variables reveal association with implantation and live birth following in vitro fertilization: A retrospective study using data from transferred human embryos
Source: PLoS One. 2020 Nov 19;15(11):e0242377. doi: 10.1371/journal.pone.0242377 (PMC7676704; doi:10.1371/journal.pone.0242377)
Supplement: S2 Table — (DOCX) [file pone.0242377.s002.docx]

**S2 Table. The relationship between BMI and KID-rate (FHB, LB) grouped in BMI quartiles.**

|  | **FHB-KID**  **No of embryos** | **BMI limit**  **(kg m^-2^)** | **FHB-KID**  **rate (%)** | **LB-KID**  **No of embryos** | **BMI limit**  **(kg m^-2^)** | **BMI limit**  **(kg m^-2^)** |
| --- | --- | --- | --- | --- | --- | --- |
| Q1 | 711 | ≤ 21.1 | 22.5 | 699 | ≤ 21.1 | 19.2 |
| Q2 | 731 | 21.2- 23.7 | 24.4* | 686 | 21.2- 23.6 | 21.1* |
| Q3 | 680 | 23.8 - 27.5 | 21.8 | 629 | 23.6 - 27.4 | 17.6 |
| Q4 | 699 | ≥ 27.5 | 18.3** | 686 | ≥ 27.5 | 14.0** |

**P* < 0.05 ***P* < 0.01

BMI quartiles : Q1,Q2,Q3 and Q4. FHB-KID: fetal heart beat implantation rate. LB-KID: live birth rate.
