# Supplementary material for: Time-lapse imaging derived morphokinetic variables reveal association with implantation and live birth following in vitro fertilization: A retrospective study using data from transferred human embryos
Source: PLoS One. 2020 Nov 19;15(11):e0242377. doi: 10.1371/journal.pone.0242377 (PMC7676704; doi:10.1371/journal.pone.0242377)
Supplement: S3 Table — Ocurrences of P < 0.001 are marked in red. (DOCX) [file pone.0242377.s003.docx]

**S3 Table. Timing of the kinetic variables from 2827 transferred embryos according to quartiles with FHB-KID rates. Ocurrences of *P* < 0.001 are marked in red.**

|  |  |  | **Q1** |  | **Q2** |  | **Q3** |  | **Q4** |
| --- | --- | --- | --- | --- | --- | --- | --- | --- | --- |
| **Variable** | **No of embryos**  **(n)** | **Limit  (Hours)** | **FHB-KID rate (%)** | **Limit  (Hours)** | **FHB-KID rate (%)** | **Limit  (Hours)** | **FHB-KID rate (%)** | **Limit  (Hours)** | **FHB-KID rate (%)** |
| tPNf | 1747 | ≤ 22.57 | 29.5*** | 22.58-24.45 | 27.7* | 24.46 -26.54 | 25.2 | ≥ 26.55 | 12.4*** |
| t2 | 2822 | ≤ 25.36 | 28.3*** | 25.37-27.20 | 25.1* | 27.21-29.42 | 21.5 | ≥ 29.43 | 12.1*** |
| VPN | 1744 | ≤ 2.34 | 20.4 | 2.35-2.67 | 29.1** | 2.68 -3.00 | 24.3 | ≥ 3.01 | 21.1 |
| t3 | 2597 | ≤ 36.06 | 26.2* | 36.07-38.39 | 27.0** | 38.40-40.80 | 24.2 | ≥ 40.81 | 15.4*** |
| cc2 | 2597 | ≤ 10.67 | 22.2 | 10.68-11.51 | 27.7** | 11.52-12.34 | 25.4 | ≥ 12.35 | 17.4*** |
| t4 | 2481 | ≤ 36.99 | 30.9*** | 34.00-39.33 | 26.6 | 39.34-41.66 | 22.3 | ≥ 41.67 | 16.0*** |
| t4 - t2 | 2479 | ≤ 11.34 | 27.1* | 11.35-12.33 | 27.6** | 12.34-13.18 | 25.3 | ≥ 13.19 | 15.7*** |
| s2 | 2479 | ≤ 0.33 | 27.9** | 0.34-0.66 | 25.0 | 0.67-1.16 | 25.8 | ≥ 1.17 | 17.0*** |

**P* < 0.05 ***P* < 0.01 ****P* < 0.001

FHB-KID: fetal heart beat - known implantation data. Morphokinetic variables grouped in quartiles: Q1, Q2, Q3 and Q4
