# Supplementary material for: Time-lapse imaging derived morphokinetic variables reveal association with implantation and live birth following in vitro fertilization: A retrospective study using data from transferred human embryos
Source: PLoS One. 2020 Nov 19;15(11):e0242377. doi: 10.1371/journal.pone.0242377 (PMC7676704; doi:10.1371/journal.pone.0242377)
Supplement: S4 Table — (DOCX) [file pone.0242377.s004.docx]

**S4 Table. AUC values to evaluate the ability of continuous TLI variables to predict LB-KID for SET and DET.**

| **TLI Variable** | **No. of embryos** | **AUC (SET)**  (LB-KID) | **No. of embryos** | **AUC (DET)**  (LB-KID) |
| --- | --- | --- | --- | --- |
| tPNf | 777 | 0.610*** | 926 | 0.630*** |
| t2 | 1163 | 0.605*** | 1601 | 0.614*** |
| VPN | 777 | 0.504 | 923 | 0.521 |
| t3 | 1100 | 0.574*** | 1442 | 0.575*** |
| cc2 | 1100 | 0.540 | 1442 | 0.537 |
| t4 | 1066 | 0.588*** | 1362 | 0.593*** |
| t4 - t2 | 1066 | 0.561** | 1360 | 0.564* |
| s2 | 1066 | 0.527 | 1360 | 0.550 |

**P* < 0.05 ***P* < 0.01 ****P* < 0.001

AUC: Area under the curve. TLI: time-lapse imaging. LB-KID: live birth. SET: single embryo transfer. DET: double embryo transfer.
